# Incessant Focal Atrial Tachycardia Leading to Tachycardiomyopathy

**DOI:** 10.7759/cureus.12770

**Published:** 2021-01-18

**Authors:** Dinkar Bhasin, Gaurav Arora, Anunay Gupta, Hermohander S Isser, Sandeep Bansal

**Affiliations:** 1 Cardiology, Vardhman Mahavir Medical College and Safdarjung Hospital, New Delhi, IND

**Keywords:** atrial tachycardia, left ventricular systolic dysfunction, tachycardiomyopathy, heart failure, supraventricular tachycardia, cardiac electrophysiology, electrocardiogram (ecg/ekg), radiofrequency ablation

## Abstract

A 22-year-old man presented with severe left ventricular (LV) dysfunction and progressive heart failure. The 12-lead electrocardiogram showed short runs of supraventricular tachycardia suggestive of focal atrial tachycardia. The patient underwent successful radiofrequency ablation. There was a complete recovery of symptoms and LV function at six months of follow-up. We discuss the importance of identifying tachycardiomyopathy as a reversible cause of heart failure.

## Introduction

All patients presenting with heart failure should undergo a diligent search for the underlying etiology [1]. This evaluation should focus on the reversible causes of heart failure. Tachycardiomyopathy is one such potentially curable cause of heart failure [2]. However, patients with heart failure often have complicating tachycardias such as atrial fibrillation (AF) and ventricular tachycardias. Hence, the identification of tachycardia as the primary cause of left ventricular (LV) dysfunction can be difficult and may be missed. The presence of an incessant arrhythmia in a young patient with LV dysfunction and no other comorbidities should raise the suspicion for tachycardiomyopathy. However, the diagnosis can only be established retrospectively once the tachycardia is treated and LV function recovers.

In the present case, a young man presented with recent-onset heart failure and was initially diagnosed to have dilated cardiomyopathy. The 12-lead electrocardiogram (ECG) showed incessant runs of supraventricular tachycardia (SVT) with a long RP interval. A careful review of the ECG helped in ascertaining the mechanism and localizing the origin of the SVT. Successful management of the tachycardia resulted in complete recovery of LV function, confirming the diagnosis as tachycardiomyopathy.

## Case presentation

**Figure 1:**
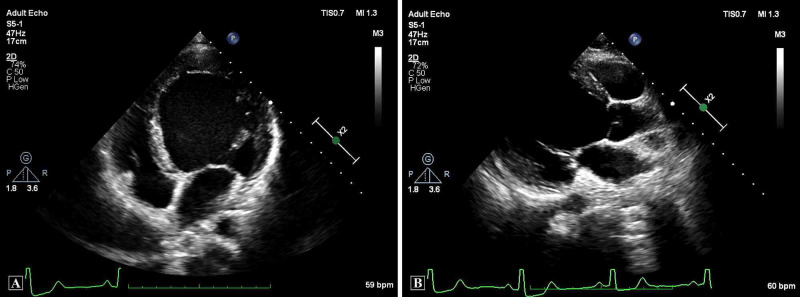
Transthoracic echocardiography at presentation. Apical four-chamber view (A) and parasternal long-axis view (B) showing dilated LV. The internal dimensions of the LV were 69 mm in diastole and 62 mm in systole. LV, left ventricular

**Figure 2:**
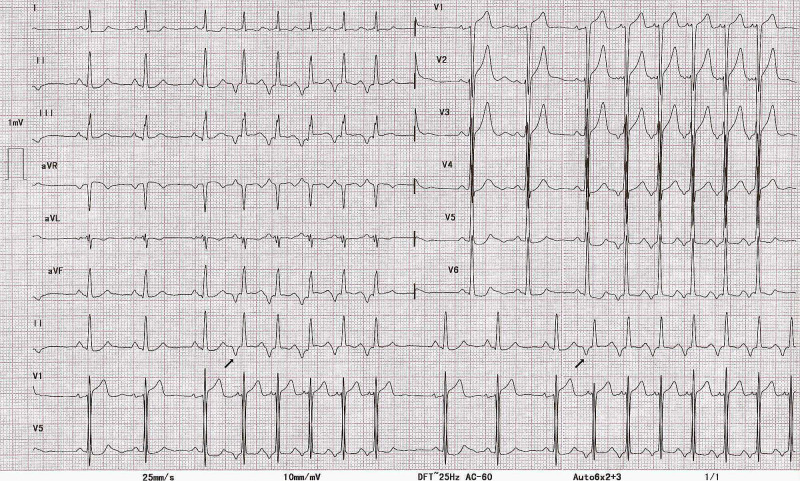
Twelve-lead ECG at presentation. There are two runs of non-sustained narrow complex tachycardia with a long RP interval and 1:1 AV relationship. The tachycardia initiates with a P-wave (arrows) without any ectopic and terminates with a QRS. The tachycardia P-wave are directed superiorly and broader than sinus P-waves. The PR interval during tachycardia is longer than sinus PR interval. ECG, electrocardiogram

A 22-year-old man presented to the out-patient department with the complaint of progressive exertional dyspnea for six months. The ECG showed global LV hypokinesia with an ejection fraction of 25%. The valve functions were normal (Figure 1, Video 1). The 12-lead ECG at presentation showed repetitive runs of narrow complex tachycardia with 1:1 AV relationship and a long RP interval (Figure 2). The tachycardia initiated with a P-wave without any ectopic and terminated with a QRS. The tachycardia P-waves had a superior axis, were broader than sinus P-waves, and the PR interval was longer compared to sinus rhythm. There was variability in tachycardia cycle length (TCL) along with varying RP and PR intervals. These ECG features were suggestive of focal atrial tachycardia (AT). Positive P-waves in lead V1 and a superior P-wave axis suggested an inferior left atrial origin of the AT.

**Video 1:** Transthoracic echocardiography at presentation. Apical four-chamber view, parasternal long-axis view, and parasternal short-axis view demonstrating LV dilatation and severe dysfunction. LV, left ventricular

**Figure 3:**
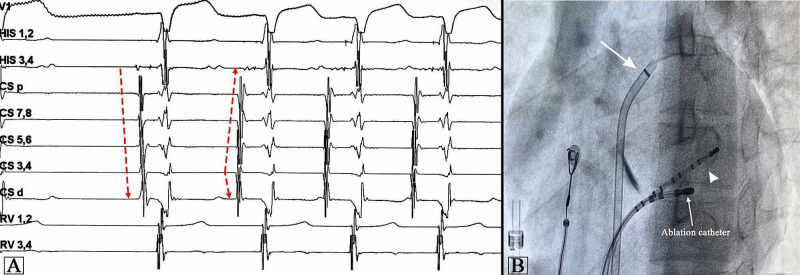
Electrophysiology study. (A) Intracardiac electrograms showing spontaneous onset of tachycardia with the earliest atrial signal at CS 3,4. (B) Fluoroscopic image in the left anterior oblique projection showing a decapolar catheter in the CS (white arrow) and sheath in LA (arrowhead). Successful ablation was done in the CS at the site of earliest atrial electrogram near the CS pole 5-6 in the CS body. CS, coronary sinus; LA, left atrium

During the electrophysiology study, there were spontaneous runs of tachycardia with the earliest atrial activation in coronary sinus (CS) 3-4 (Figure 3A). No ventriculoatrial conduction was present at baseline or during isoproterenol infusion, excluding AV node-dependent tachycardias such as atrioventricular re-entrant tachycardia (AVRT) and atrioventricular nodal re-entrant tachycardia (AVNRT). We mapped the left atrium (LA) after obtaining trans-septal access, but the activation signals were late. We then mapped the CS, and the earliest A signals relative to the surface P-waves were observed in the mid-CS near the CS pole 5-6 (Figure 3B). The tachycardia terminated within five seconds of the first ablation and could not be induced thereafter. The patient was discharged on medical therapy for heart failure, including angiotensin-converting enzyme inhibitors, beta-blockers, and diuretics.

**Figure 4:**
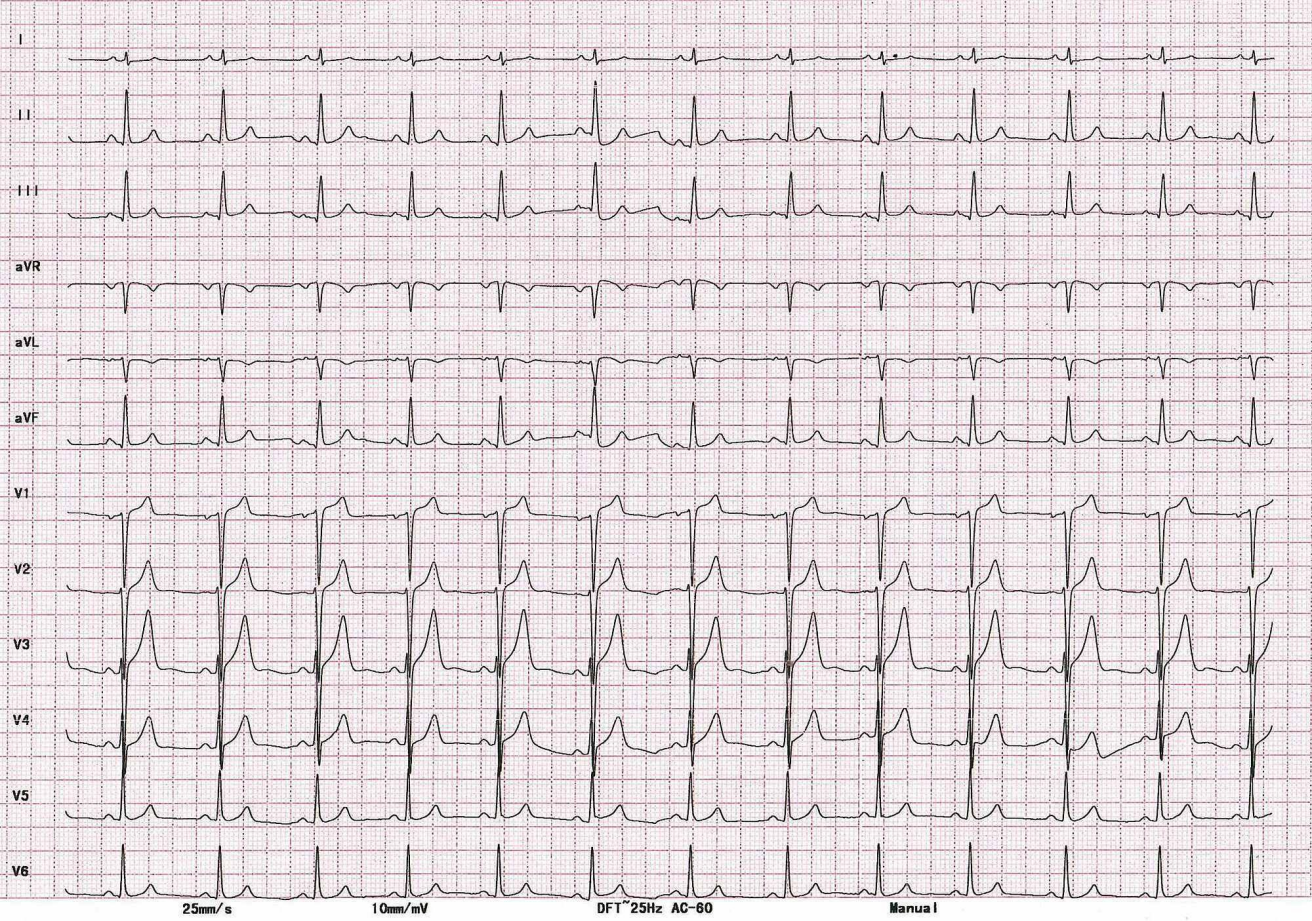
Twelve-lead ECG after six months of follow-up showing normal sinus rhythm. ECG, electrocardiogram

**Figure 5:**
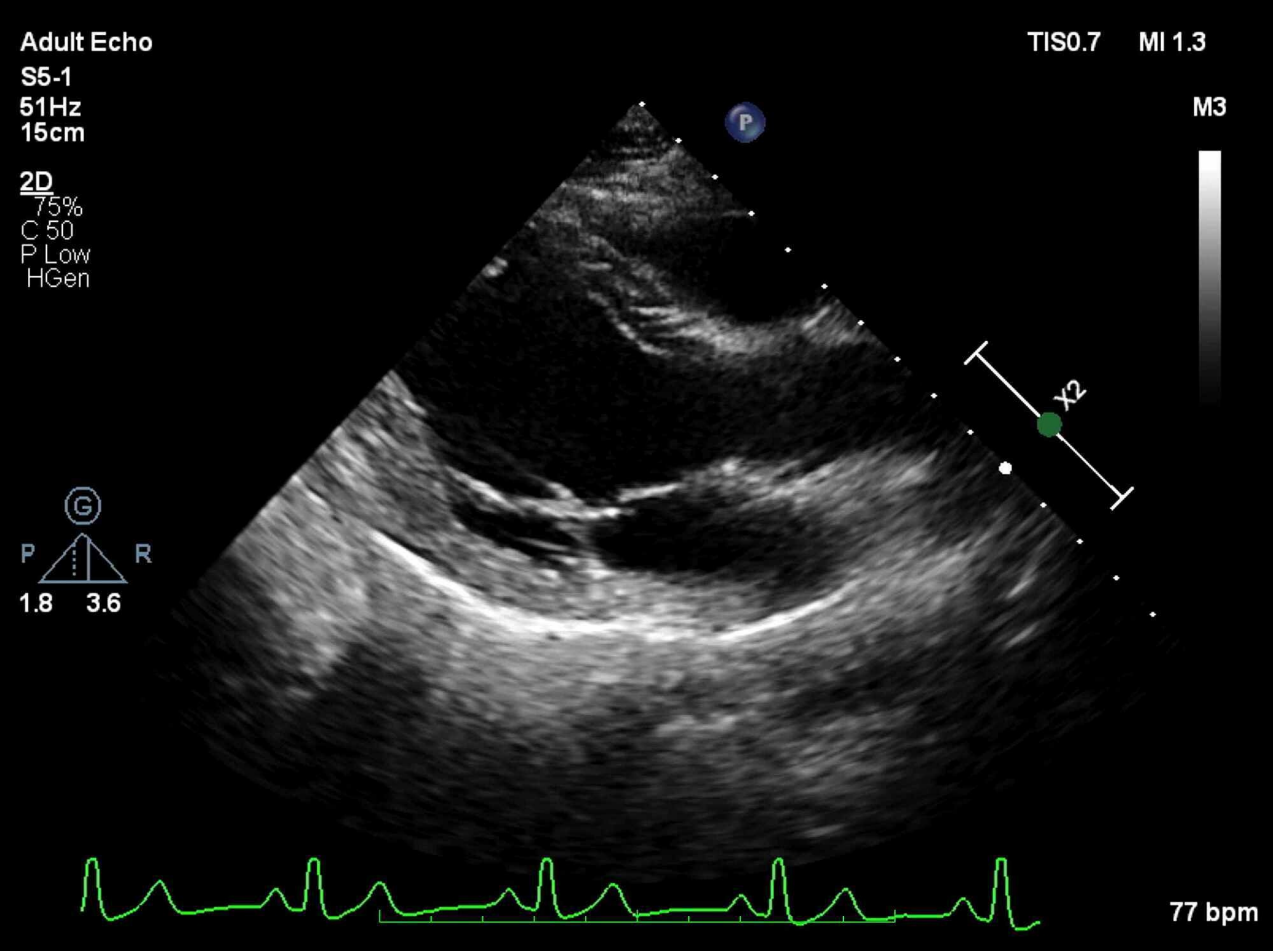
Transthoracic echocardiography at six months of follow-up. Parasternal long-axis view showing normal-sized left ventricle. The LV internal dimensions were 54 mm in diastole and 39 mm in systole. LV, left ventricular

After six months of follow-up, the patient was asymptomatic. The electrocardiogram was normal (Figure 4), and there was complete recovery of LV function (Figure 5, Video 2). The recovery of LV function after successful ablation of tachycardia confirmed the retrospective diagnosis as tachycardiomyopathy.

**Video 2:** Transthoracic echocardiography at six months of follow-up. Parasternal long-axis view and parasternal short-axis view demonstrating normal LV systolic function. LV, left ventricular

## Discussion

AF and frequent premature ventricular complexes (PVC) are the most common arrhythmias associated with tachycardiomyopathy [2]. SVTs such as AT and AVNRT when incessant can also lead to heart failure [3]. As these tachycardias may not be persistent but occur in short runs, they can be missed in the standard 12-lead ECG. Further, incessant ATs, which lead to tachycardiomyopathy, usually occur at a slower rate and may cause minimal symptoms [4]. The first presentation can be with heart failure, and the tachycardia may be misdiagnosed as sinus tachycardia if the differences in P-wave morphology and axis are not carefully appreciated.

Tachycardiomyopathy should be suspected in all patients with LV dysfunction and an elevated mean heart rate on 24-hour monitoring, frequent PVCs, and AF [2]. In patients without obvious tachycardia on a standard 12-lead ECG, ambulatory ECG monitoring for two weeks should be considered to reliably exclude tachycardiomyopathy [2]. In addition to the diagnosis of the arrhythmia, ambulatory ECG monitoring helps in estimating the burden. In the present case, there were runs of long RP SVT in the ECG. Careful analysis of the tachycardia features helped in reaching the correct diagnosis. When the origin and termination of an SVT are clearly visible in the ECG, precise diagnosis is often possible.

The differential diagnosis of an SVT with a long RP includes focal AT, atypical AVNRT (fast-slow, slow-slow), and orthodromic AVRT with a slow, decrementally conducting accessory pathway [5]. While atypical AVNRT (aAVNRT) rarely causes LV dysfunction, AT and AVRT can cause an incessant tachycardia leading to tachycardiomyopathy [3]. The AVRT most commonly implicated in such a case is permanent junctional re-entrant tachycardia (PJRT) with the pathway located in the posterior septum. Differentiating between these tachycardias on a surface ECG may be difficult. However, certain ECG characteristics such as tachycardia onset, termination, and spontaneous variations can help make the distinction [5].

Focal ATs start either with an ectopic beat or spontaneously. PJRT initiates during sinus rhythm after shortening of sinus cycle length, and AVNRT starts with an ectopic beat [6]. In the present case, the tachycardia started after lengthening of the preceding sinus beat, making PJRT unlikely. It is difficult to exclude that the first P-wave initiating the tachycardia is not ectopic. However, the same P-wave morphology throughout the run makes this less probable. All three forms of SVT can terminate spontaneously with a QRS, which usually does not help in differentiating between them; nonetheless, focal AT is more likely to terminate with QRS.

Spontaneous variation in the different electrocardiographic intervals can provide valuable insights into the mechanism of SVT. Wide variations in TCL occur in AF or multifocal AT. Re-entrant tachycardia may also have subtle variation in TCL. In most re-entrant tachycardias involving the AV node, the RP interval is generally constant. However, in aAVNRT and PJRT, the RP interval may vary because of decremental conduction over the slow AV nodal pathway and accessory pathway, respectively. In PJRT, the changes in RR interval precede the changes in PP interval, while in focal AT and aAVNRT, changes in PP interval precede the changes in RR interval. In the present case, there was significant variability in TCL due to varying RP and PR intervals favoring AT as the likely diagnosis (Figure 3).

The surface ECG can help in identifying the site of origin of focal AT. Kistler et al. suggested an algorithm based on lead V1 to localize focal AT with high sensitivity and specificity; however, it is difficult to remember [3]. A simplified algorithm using the P-wave morphology in leads V1 and I and the inferior leads helps in localizing the origin of the AT [7]. A positive P-wave in lead V1 and a biphasic or negative P-wave in lead I suggest a tachycardia originating from the LA. A positive P-wave morphology in lead I and negative in V1 suggest a right atrial origin of the tachycardia. Leads II, III, and lead aVF helps distinguish superior from inferior atrial origin of the tachycardia [7].

All patients with tachycardiomyopathy should receive medical therapy for heart failure, including beta-blockers and renin-angiotensin inhibitors. The definitive treatment is radiofrequency ablation of the tachyarrhythmia. Drug therapy with amiodarone has moderate efficacy but significant long-term side effects. Therapy with ivabradine has shown benefit in ATs arising from atrial appendages and can be used while awaiting ablation [8].

## Conclusions

Tachycardiomyopathy is a reversible cause of heart failure. This case underscores the importance of identifying SVTs that can lead to tachycardiomyopathy. Careful analysis of the surface ECG helps in the diagnosis of the tachycardia. This is particularly rewarding when both onset and termination of the tachycardia can be seen on 12-lead ECG.
